# Non-lethal effects of the predator *Meganyctiphanes norvegica* and influence of seasonal photoperiod and food availability on the diel feeding behaviour of the copepod *Centropages typicus*

**DOI:** 10.1093/plankt/fbaa051

**Published:** 2020-11-03

**Authors:** Manuel Olivares, Peter Tiselius, Albert Calbet, Enric Saiz

**Affiliations:** INSTITUT DE CIéNCIES DEL MAR (ICM, CSIC), PG. MARíTIM DE LA BARCELONETA 37-49, E-08003 BARCELONA, SPAIN; DEPARTMENT OF BIOLOGICAL AND ENVIRONMENTAL SCIENCES – KRISTINEBERG, UNIVERSITY OF GOTHENBURG, KRISTINEBERG 566, 45178 FISKEBÄCKSKIL, SWEDEN; INSTITUT DE CIéNCIES DEL MAR (ICM, CSIC), PG. MARíTIM DE LA BARCELONETA 37-49, E-08003 BARCELONA, SPAIN; INSTITUT DE CIéNCIES DEL MAR (ICM, CSIC), PG. MARíTIM DE LA BARCELONETA 37-49, E-08003 BARCELONA, SPAIN

**Keywords:** diel rhythms, predator–prey interactions, zooplankton, krill, faecal pellets

## Abstract

Predators can induce changes in the diel activity patterns of marine copepods. Besides vertical migration, diel feeding rhythms have been suggested as an antipredator phenotypic response. We conducted experiments to assess the non-lethal direct effects of the predator *Meganyctiphanes norvegica* (northern krill) on the diel feeding patterns of the calanoid copepod *Centropages typicus*. We also analysed the influence of seasonal photoperiod and prey availability on the intensity of copepod feeding rhythms. We did not detect any large effect of krill presence on the diel feeding behaviour of copepods, either in day-night differences or total daily ingestions. Seasonal photoperiod and prey availability, however, significantly affected the magnitude of copepod feeding cycles, with larger diel differences in shorter days and at lower prey concentrations. Therefore, the role of non-lethal direct effects of predators on the diel feeding activity of marine copepods remain debatable and might not be as relevant as in freshwater zooplankton.

## INTRODUCTION

Predation threat can trigger a wide variety of responses in animals, such as changes in morphology, physiology and/or behaviour (Lima, 1998; Agrawal, 2001). These predator-induced phenotypic changes have evolved to increase the animal’s survival chances, but also bring certain fitness costs in terms of energy acquisition and resource allocation (Sih, 1980; Lima and Dill, 1990; Preisser *et al.*, 2005). Thus, predators can have negative impacts on prey populations not only through direct predation (consumptive effects), but also through the so-called non-consumptive (or non-lethal) effects. Non-lethal effects of predation can even represent a higher cost for prey demography than predation itself (Preisser *et al.*, 2005), and certainly have important ecological implications regarding community dynamics (Werner and Peacor, 2003; Schmitz *et al.*, 2004).

Within marine communities, copepods are a vital link between primary producers and fish (Runge, 1988) and typically account for the highest abundance and biomass among mesozooplankton (Longhurst, 1985). Therefore, non-lethal effects of predators on copepod populations can translate into important cascading effects in marine food webs (van Someren Gréve *et al.*, 2019). Copepod responses to increasing predation risk include changes in their foraging time, swimming speed and reproductive output (Saiz *et al.*, 1993; van Duren and Videler, 1996; Lasley-Rasher and Yen, 2012; Heuschele *et al.*, 2014). Of particular relevance is how predation risk can alter copepod diel behaviour. For instance, predation threat appears to be the major driver of diel vertical migrations in marine copepods (Frost, 1988; Ohman, 1990; Bollens and Frost, 1991; Hylander and Hansson, 2013). Migrant copepods typically stay in food-enriched upper waters at night, and move to deeper, darker layers during the daytime to avoid visual predation.

Besides vertical migration, copepods frequently show other diel activity rhythms involving their feeding, spawning and moulting patterns (Ohman, 1988). About the former, copepods generally show higher feeding activity at night (Atkinson *et al.*, 1992; Dagg *et al.*, 1998). Nocturnal feeding is usually coupled to vertical migrations, but this feeding behaviour is not necessarily a consequence of staying in food-enriched upper layers at night. In fact, rhythmic feeding may also appear in non-migratory copepods (Hayward, 1980; Head *et al.*, 1985). Therefore, diel feeding rhythms of marine copepods might confer an adaptive advantage that is independent of vertical migration.

Feeding in copepods implies higher motility and conspicuousness, which increases their detectability and predation vulnerability (Tsuda *et al.*, 1998; Uttieri *et al.*, 2013; Kiørboe *et al.*, 2014). This especially applies to daylight hours when copepods are more susceptible to visual predation (Tsuda *et al.*, 1998; Torgersen, 2001). Thus, feeding rhythms (i.e. lower daytime activity) have been traditionally considered an antipredator strategy in copepods (Ohman, 1988). Bollens and Stearns (1992) and Cieri and Stearns (1999) found that the planktonic copepods *Acartia tonsa* and *Acartia hudsonica* showed a lower daytime gut fullness when exposed to fish or fish exudates. However, other studies have not found any effect of predation threat on the feeding behaviour of marine copepods (Kiørboe *et al.*, 2018; Olivares *et al.*, 2020). Hence, the predator effects on copepod feeding rhythms still remain unclear. Most former research on this topic relied on predator exudates as predatory signals, even though marine copepods are known to respond to hydromechanical cues generated by predators (Kiørboe *et al.*, 1999; Hwang and Strickler, 2001; Buskey *et al.*, 2011). In this respect, further experiments with copepods exposed to freely swimming predators are necessary to detect predator-induced responses that are not only chemically triggered (e.g. Saiz *et al.* 1993; Tiselius *et al*. 1997).

The intensity of feeding-related antipredator responses of copepods (e.g. feeding rhythms) can depend on other factors besides predation threat. Copepod diel rhythms can show great seasonal variations (Durbin *et al.*, 1995; Irigoien *et al.*, 1998). These seasonal differences could be attributed to changes in the relative length of daylight periods associated to a higher predation threat (Lima and Bednekoff, 1999). Also, copepods can adapt their foraging behaviour to changing food conditions that affect their risk of being predated (Tiselius *et al.*, 1997; Visser, 2007; van Someren Gréve *et al.*, 2019). However, the effect of increasing food availability on copepod feeding rhythms is controversial. For instance, Hassett and Blades-Eckelbarger (1995) found that day-night differences in copepod feeding activity became larger at lower prey concentrations, whereas Calbet *et al.* (1999) reported that lower food concentrations did not affect or led to weaker diel feeding rhythms in some species.

The main goal of our study was to determine the non-lethal effects of predators on the diel feeding behaviour of marine copepods. Additionally, we also analysed the influence of seasonal photoperiod and prey availability on the magnitude of copepod feeding rhythms. We conducted laboratory experiments with the calanoid copepod *Centropages typicus* and the krill *Meganyctiphanes norvegica* as predators. *M. norvegica* acts as a key predator and grazer in pelagic communities of the North Atlantic with a preference for large and medium-sized copepods (Beyer, 1992; Båmstedt and Karlson, 1998; Agersted and Nielsen, 2016), and is a fundamental prey item for larger fish, squids and whales (Schmidt, 2010; Simard and Harvey, 2010; Suca *et al.*, 2018).

## METHOD

### Experimental organisms

The heterotrophic dinoflagellate *Oxyrrhis marina*, the calanoid copepod *Centropages typicus* and the euphausiid *Meganyctiphanes norvegica* were used for experiments in summer 2018 and autumn 2019.

*O. marina* was grown in 0.5-μm filtered seawater at 18 ± 0.5°C and fed daily with the cryptophyte *Rhodomonas salina*. *R. salina* was grown in B medium (experiments in 2018, Hansen (1989)) or f/2 medium (experiments in 2019, Guillard (1983)). The cultures of *O. marina* were not fed for 48 h before experiments to ensure the absence of *R. salina* cells during incubations.

Copepods were collected in the Gullmar Fjord (58° 15.7' N, 11° 26.7′ E, Sweden) using a 250-μm mesh plankton net. In the laboratory adult females of *C. typicus* were isolated using a pipette and kept in 8-L polycarbonate tanks with filtered seawater and food (*O. marina*, > 4 ppm). The sorted copepods were maintained at 14.5 ± 0.5°C under a photoperiod that simulated natural light conditions: 16 h: 8 h light: dark in summer, and 10 h: 14 h light: dark in autumn.

Krill (*M. norvegica*) were collected in the deepest part of the fjord (58° 19.0' N, 11° 32.6′ E) using an Isaacs-Kidd Midwater Trawl. Upon arrival at the station, the specimens were transferred to a 300-L glass fibre flow-through tank at 10°C and turnover rate 450 L h^−1^. Krill were kept in constant darkness and fed daily with freshly collected zooplankton from the fjord.

### Experimental set-up

Experiments consisted of day and night incubations of copepods (*C. typicus*) feeding on *O. marina* in the absence and the presence of predators (krill *M. norvegica*). Before incubations, copepods were collected from their maintenance tanks using a 200-μm mesh sieve and placed in filtered seawater for ca. 1.5 h to allow gut evacuation. The cell concentration of *O. marina* stock culture was determined with a Z Series Coulter Counter. About 8 to 10 bottles were filled with filtered seawater and *O. marina* was added to each bottle to obtain final prey concentrations of either 5.5–7 ppm (ca. 1900–2700 cells mL^−1^; high food; five experiments) or 1.0 ppm (ca. 250 cells mL^−1^; low food; one experiment). The bottle volumes and *O. marina* concentrations in the experiments are shown in Table I. Four of the bottles were used as control bottles (*O. marina* + copepods) and four to six bottles as experimental bottles (*O. marina* + copepods + krill). A total of 30 copepods and one krill were added to each experimental bottle using, respectively, a wide-mouth pipette and an aquarium fish net (except for one experiment, with only 20 copepods per bottle). The copepod densities in the bottles (7.5–13 cop L^−1^) were comparable to those that can be found in the Gullmar Fjord (Vargas *et al.*, 2002; Tönnesson and Tiselius, 2005). In the case of krill, the experimental densities (0.25–0.4 ind L^−1^) were higher than the typical average densities of *M. norvegica* in nature (Onsrud and Kaartvedt, 1998; Tarling *et al.*, 1998), but fell within the range of densities reported for dense krill swarms (Nicol, 1986; Kaartvedt *et al.*, 2005). The bottles were then incubated for 8.5–11.5 h in a temperature-controlled room at 14.5 ±  0.5°C and under the seasonal photoperiod specified before (Table I). The bottles were lit from the side to diminish vertical heterogeneity in the distribution of *O. marina* and *C. typicus* due to small-scale migrations during incubations (Alcaraz *et al.*, 2007; Bochdansky *et al.*, 2010; Bollens *et al.*, 2011). After the incubations, the contents of the bottles were sieved through a 200-μm mesh to collect copepods and krill*,* and then through a 20-μm mesh to collect copepod faecal pellets. The survival of copepods and krill was checked and the number of dead copepods was noted. The bottles with dead krill were discarded for data analysis (2 out of 30 bottles). The length of krill specimens was measured with a ruler. Copepods and faecal pellets were preserved with Lugol’s solution for number and size determination. Photos of 20 copepods and 60–70 faecal pellets were taken per treatment (i.e. with and without krill), and the prosome length of copepods and the length and width of faecal pellets were measured with the software ImageJ (Schneider *et al.*, 2012). *O. marina* size was obtained from Coulter Counter data registered at the beginning of the incubations.

**Table I:** Temperature, light conditions, concentrations of prey (*Oxyrrhis marina*) and copepods (*Centropages typicus*) and bottle volumes used in the experiments. Mean ± SD are provided

Experiment	Date	Temperature (°C)	Photoperiod (day: night)	Irradiance (μmol photons m^−2^ s^−1^)	Prey conc. (ppm)	Copepods per bottle	Bottle volume (mL)
1	12 Aug	14.7 ± 0.23	16 h: 8 h	2.2 ± 0.21	5.5	20	2 300
2	16 Aug	14.7 ± 0.23	16 h: 8 h	2.2 ± 0.21	7.0	30	2 300
3	24 Aug	14.7 ± 0.23	16 h: 8 h	2.2 ± 0.21	6.0	30	2 300
4	18 Oct	14.4 ± 0.11	10 h:14 h	1.7 ± 0.33	6.5	30	4 000
5	25 Oct	14.4 ± 0.11	10 h:14 h	1.7 ± 0.33	1.0	30	4 000
6	28 Oct	14.4 ± 0.11	10 h:14 h	1.7 ± 0.33	6.5	30	4 000

The number of replicates was determined based on power calculations and published data on variability. For the predation by krill, Lass *et al.* (2001, Fig. 7C) reported day/night differences in gut fullness determined from the number of copepod mandibles in the gut. The krill contained 39% more mandibles at night and the standard deviation of the gut fullness was ~25% of the mean. To detect a similar difference with a power = 0.8 required 17 replicates from each of day and night (df = 32) in our study. For the faecal pellet production, we used the clearance rates in Calbet *et al.* (1999, Fig. 1) for *C. typicus*, which were 75% higher at night and with a standard deviation ~40% of the mean. With a standard deviation = 40% and a predicted difference of 50% between day and night, a design with 11 replicates from each of day and night (df = 20) was required for a power = 0.8. Since it was not possible to run all replicates in one experiment, the entire experiments were repeated three times in summer and in autumn.

### Pilot experiments—correction factors for data analysis

The krill *M. norvegica* may feed on small-sized phytoplankton and microzooplankton cells (Agersted and Nielsen, 2016), as well as on detritus and sediments (Youngbluth *et al.*, 1989; Lass *et al.*, 2001). Therefore, pilot experiments were conducted to account for any potential effect of krill on *O. marina* concentration and/or copepod faecal pellet accumulation in the incubations.

To check for krill grazing on *O. marina*, 11 bottles of 4 L were filled with acclimatized filtered seawater and adjusted to 1 ppm of *O. marina* following the same methodology as in the main experiments. Among the 11 bottles, three bottles were used as initial bottles (only *O. marina*), four as control bottles (only *O. marina*) and four as experimental bottles (*O. marina* and one krill). The organisms were added to the bottles as described in the previous section. Control and experimental bottles were then incubated for 10 h under the same conditions as in the main experiments, and initial and final *O. marina* concentrations were measured with a Coulter counter.

Two incubations were carried out to determine krill grazing on copepod faecal pellets. Six bottles (first incubation) or 10 bottles (second incubation) of 4 L were filled with acclimatized filtered seawater. Half of the bottles served as control bottles (only faecal pellets) and the other half as experimental bottles (faecal pellets and one krill). Faecal pellets were collected from copepod tanks by siphoning the tank bottoms and removing copepods with a 200-μm mesh. The faecal pellet concentration was estimated by counting three subsamples, and then aliquots containing around 350 faecal pellets were added to each bottle. Krill were transferred to the experimental bottles using an aquarium net. The bottles were then incubated for 10 h in the same conditions as in the main experiments. At the end of the incubations, the krill and the faecal pellets were collected using a 20-μm mesh. The faecal pellets were fixed in acidic Lugol’s solution for counting and size determination. A total of 60 faecal pellets per treatment (i.e. with and without krill) were photographed, and length and width measurements were conducted with ImageJ (Schneider *et al.*, 2012).

### Data analysis

The feeding activity of copepods was estimated based on their faecal pellet production rates (Nejstgaard *et al.*, 2001; Besiktepe and Dam, 2002). The average pellet volumes were calculated assuming an ellipsoidal shape. Gut evacuation times of copepods (20 min at 14°C, Irigoien (1998)) were subtracted from incubation times because copepod guts were empty before incubations. In the experimental bottles where krill actively predated on copepods, the average number of copepods during incubation was calculated assuming an exponential decrease of copepod abundance following the equations in Frost (1972).

The pilot experiments showed that in 10-h incubations the *O. marina* concentrations in the bottles did not change regardless of the absence or the presence of krill (two-tailed Student’s t-tests, *P* > 0.05). However, krill removed 15% of copepod faecal pellets during incubations (randomized block design (RBD) analysis of variance (ANOVA), F(1,13) = 10.76, *P* < 0.01). Thus, faecal pellet production rates of copepods were corrected assuming a pellet removal by krill of 1.5% per hour.

After data correction, RBD ANOVA tests with experiment as block factor were conducted to check for significant effects of the factors day/night and absence/presence of predator (krill) on copepod pellet production rates. RBD ANOVAs were applied to each set of experiments with the same photoperiod and prey concentration (i.e. experiments in summer at high food, and experiments in autumn at high food, Table I). For the only experiment at low food availability (1 ppm), a two-way ANOVA was used instead. Additionally, a two-way ANOVA was conducted to check for significant differences between seasonal photoperiod (16:8 h *vs* 10:14 h) and prey availability (high *vs* low) in the magnitude of copepod feeding rhythms (i.e. night/day ratios of pellet production rates). Finally, a two-way ANOVA was applied to krill predation rates to test significant differences between day and night, and between seasons (i.e. photoperiod). All datasets passed normality and homoscedasticity assumptions according to Shapiro–Wilk and Brown-Forsythe tests, respectively.

## RESULTS

**Fig. 1:**
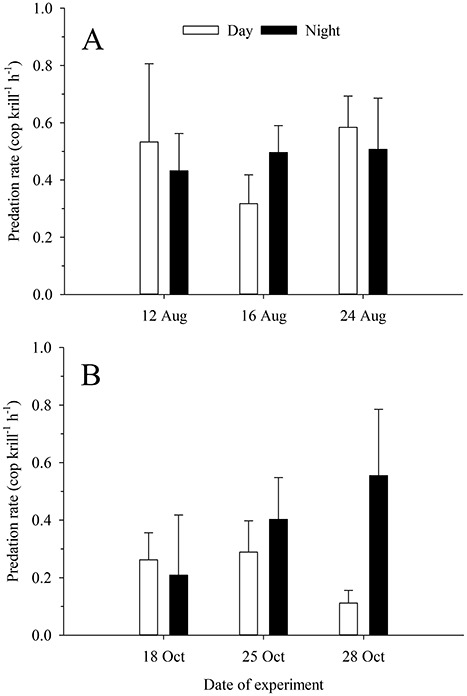
Predation rates of the krill *Meganyctiphanes norvegica* (mean ± SE, n = 4–6) on the copepod *Centropages typicus* in experiments conducted in (A) summer (16 h:8 h day: night cycle) and (B) autumn (10 h:14 h day: night cycle).

The krill *Meganyctiphanes norvegica* was actively feeding on copepods in our experiments. The average predation rates ranged 0.1–0.6 cop krill^−1^ h^−1^ and no significant differences were detected between day and night, or between seasons (two-way ANOVA, F(1,54) = 1.51 and *P* > 0.05 for day/night, F(1,54) = 3.61 and *P* > 0.05 for photoperiod; Fig. 1). The interaction between factors was also not significant (two-way ANOVA, F(1,54) = 1.21, *P* > 0.05).

**Fig. 2:**
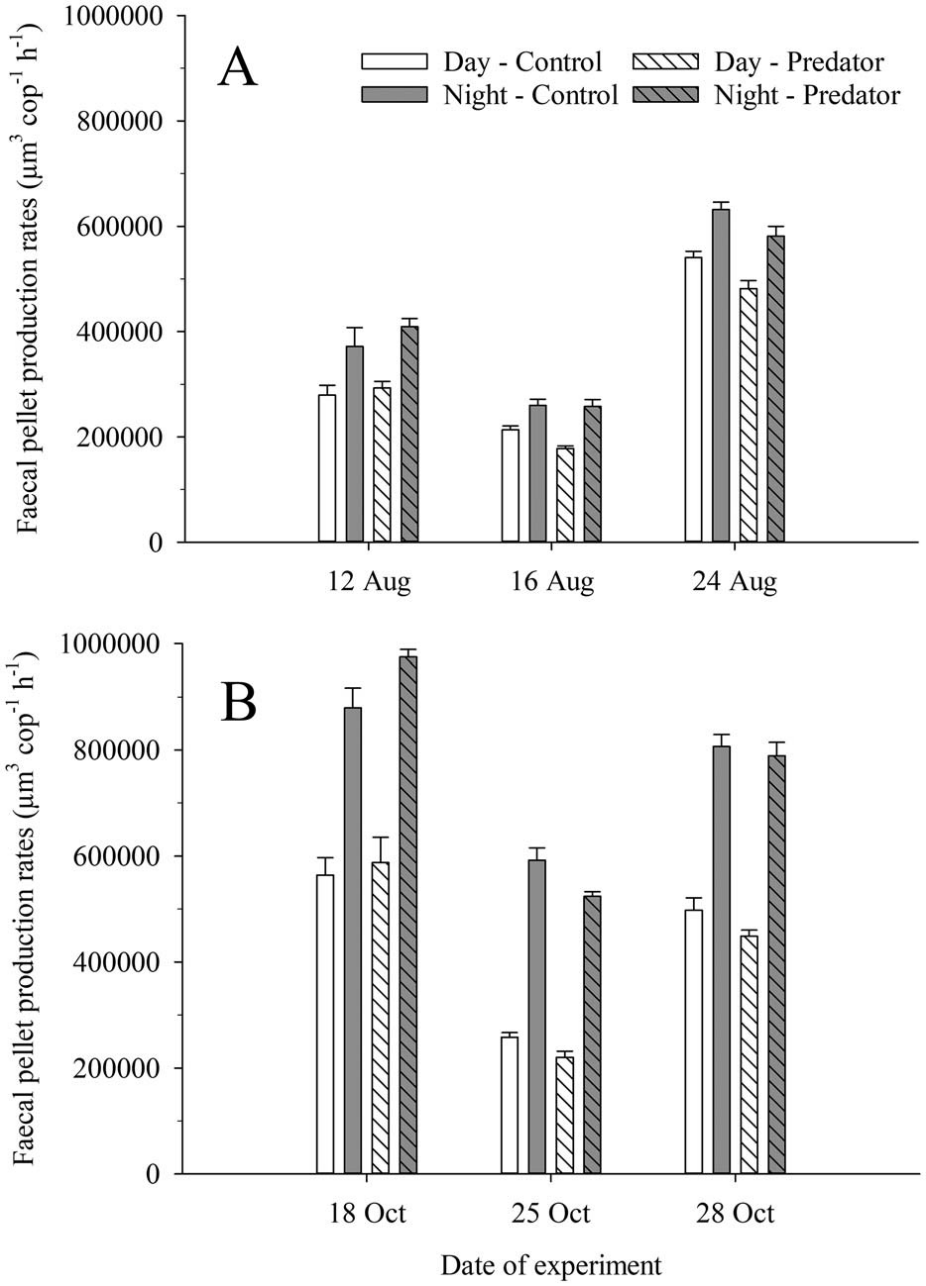
Day and night faecal pellet production rates (mean ± SE, n = 4–6) of *Centropages typicus* feeding on *Oxyrrhis marina* in the absence of predator (“Control”) and in the presence of the predator *Meganyctiphanes norvegica* (“Predator”). Data from experiments in (A) summer (16 h:8 h day: night cycle) and (B) autumn (10 h:14 h day: night cycle) are shown. Notice that *O. marina* concentrations were 1 ppm on 25 October, and 5.5–7.0 ppm in the other experiments.

Table II shows the sizes of copepods and prey, as well as the faecal pellet production rates of copepods and the pellet volumes in the incubations. All the krill specimens had a body length of ca. 40 mm. The pellet production rates of copepods were significantly higher at night in all the experiments (summer at high food: RBD ANOVA, F(1,50) = 70.63, *P* < 0.001; autumn at high food: RBD ANOVA, F(1,27) = 226.82, *P* < 0.001; autumn at low food: two-way ANOVA, F(1,13) = 495.75, *P* < 0.001; Fig. 2). The presence of predator (krill) did not have any significant effect on pellet production rates in any of the experiments at high food (summer: RBD ANOVA, F(1,50) = 3.15, *P* > 0.05; autumn: RBD ANOVA, F(1,27) = 0.04, *P* > 0.05; Fig. 2), but had a significant effect in the experiment on 25 October at low food (two-way ANOVA, F(1,13) = 13.63, *P* < 0.01; Fig. 2). However, in this last case krill caused a decrease of only 12% in the daily production of faecal pellets by copepods. No significant interactions between the factors day/night and absence/presence of predator were found (summer at high food: RBD ANOVA, F(1,50) = 0.91, *P* > 0.05; autumn at high food: RBD ANOVA, F(1,27) = 0.73, *P* > 0.05; autumn at low food: two-way ANOVA, F(1,13) = 1.19, *P* > 0.05).

**Table II:** Sizes of copepods (*Centropages typicus*) and prey (*Oxyrrhis marina*), faecal pellet production rates of copepods and pellet volumes in incubations without predator (“Control”) and with the predator *Meganyctiphanes norvegica* (“Predator”). Mean ± SE are shown. ESD: equivalent spherical diameter. n.d.: not determined

				Control		Predator	
Date of experiment	Time period	Copepod size (μm)	Prey size (ESD, μm^3^)	Pellet production (pellets cop^−1^ h^−1^)	Pellet volume (μm^3^)	Pellet production (pellets cop^−1^ h^−1^)	Pellet volume (μm^3^)
12 Aug	Day	n.d.	16.2 ± 0.05	1.5 ± 0.10	183 462 ± 16 954	1.5 ± 0.07	191 441 ± 10 065
	Night	n.d.	16.0 ± 0.08	1.8 ± 0.18	203 021 ± 12 497	1.8 ± 0.07	223 268 ± 10 884
16 Aug	Day	1 182 ± 8.4	17.6 ± 0.03	1.3 ± 0.04	170 313 ± 12 730	1.0 ± 0.03	171 732 ± 12 338
	Night	1 168 ± 8.6	16.6 ± 0.01	1.3 ± 0.06	199 182 ± 8 950	1.4 ± 0.07	189 389 ± 8 527
24 Aug	Day	1 164 ± 14.3	16.5 ± 0.01	1.9 ± 0.04	288 239 ± 11 924	1.8 ± 0.06	268 105 ± 10 945
	Night	1 186 ± 10.3	16.4 ± 0.03	2.1 ± 0.05	298 799 ± 16 467	1.9 ± 0.06	311 400 ± 22 596
18 Oct	Day	1 256 ± 10.7	19.2 ± 0.03	1.9 ± 0.11	289 399 ± 12 370	1.9 ± 0.16	305 818 ± 13 861
	Night	1 225 ± 13.8	18.1 ± 0.02	2.2 ± 0.09	400 043 ± 22 237	2.3 ± 0.03	428 394 ± 24 822
25 Oct	Day	1 203 ± 10.9	20.4 ± 0.04	1.2 ± 0.04	220 836 ± 13 482	1.2 ± 0.06	189 468 ± 10 139
	Night	1 205 ± 14.6	19.3 ± 0.08	1.8 ± 0.07	336 411 ± 19 286	1.6 ± 0.03	331 894 ± 19 619
28 Oct	Day	1 203 ± 10.6	17.4 ± 0.04	1.7 ± 0.08	290 791 ± 18 121	1.5 ± 0.04	303 520 ± 22 506
	Night	1 212 ± 14.5	17.0 ± 0.02	1.8 ± 0.05	443 893 ± 24 626	1.9 ± 0.06	412 872 ± 22 900

The intensity of copepod feeding rhythms (i.e. night/day ratios of pellet production rates) were significantly different between seasonal photoperiods (two-way ANOVA, F(1,9) = 29.97, *P* < 0.001) and between food conditions (two-way ANOVA, F(1,9) = 62.58, *P* < 0.001). The night/day ratios of pellet production were 1.2–1.4 in summer at high prey concentration, 1.6–1.8 in autumn at high prey concentration, and 2.3–2.4 in autumn at low prey concentration (Fig. 2).

## DISCUSSION

Previous studies linked feeding rhythms of wild copepods to nocturnal forays into food-enriched upper layers during diel vertical migrations (Baars and Oosterhuis, 1984; Simard *et al.*, 1985; Besiktepe *et al.*, 2005). In our experiments, given the bottle sizes, we did not expect any relevant light-induced spatial heterogeneity in the distribution of the copepod prey *Oxyrrhis marina* (see Methods section) that could not be overcome by the swimming activity and the prey detection capability of *Centropages typicus* (Tiselius and Jonsson, 1990; Bundy *et al.*, 1993; Gonçalves and Kiørboe, 2015). Therefore, our study suggests that the rhythmic feeding behaviour of the copepod *C. typicus* might have an adaptive significance itself, which seems independent of migratory behaviour or changing food conditions (Head *et al.*, 1985; Durbin *et al.*, 1990). As evidenced in our experiments, such rhythms do not necessarily imply the complete cessation of feeding during the daytime (*sensu*  Dagg *et al.* (1998)), but a lower foraging activity during daylight hours (Atkinson *et al.*, 1996; Olivares *et al.*, 2020).

The diel activity patterns of marine copepods can change upon the presence of predators (Ohman, 1988; Bollens and Frost, 1989; Bollens and Stearns, 1992). The krill *Meganyctiphanes norvegica* exert an important predatory pressure on copepod populations in the Northeast Atlantic (Beyer, 1992; Båmstedt and Karlson, 1998; Onsrud and Kaartvedt, 1998) and can affect their vertical migration patterns (Tarling *et al.*, 2002). Because the krill densities in our experiments were higher than typical average abundances in nature (Onsrud and Kaartvedt, 1998; Tarling *et al.*, 1998), we would expect the predator-induced responses of copepods to have been maximized. Still, we did not detect any large effect of krill presence on the feeding behaviour of copepods, either in diel feeding patterns or daily food intake. In all the cases the presence of krill barely affected the mean daily productions of copepod faecal pellets (from −8.7 to 12.2%), and such small differences proved to be statistically significant only in one out of the six experiments. Given the low variability among replicates in the faecal pellet production rates (median of coefficients of variation: 7.8%), any undetected effect of krill on copepod feeding activity was small. Thus, the non-lethal direct effects of predators on the feeding activity of marine copepods, including diel feeding rhythms, could be limited and/or depend on type of predator (Ohman, 1990).

In our study, *M. norvegica* showed predation rates that were highly variable among individuals. Such a flexible feeding behaviour has been previously observed in other experimental studies with *M. norvegica* predating on copepods (McClatchie, 1985; Båmstedt and Karlson, 1998; Agersted and Nielsen, 2016). We did not detect differences between day and night or between seasons in krill predation rates, but given the high variability of predation rates and the sample size in our experiments, we cannot discard that certain diel or seasonal differences in the feeding activity of krill could have been unnoticed (Torgersen, 2001). Our design was based on a power = 0.8 and standard deviations of 25–40% of the mean, but we observed a much higher variability than anticipated (median = 94% of the mean). It is also possible that the diurnal predation rates of *M. norvegica* could have been enhanced if copepods had been feeding on a more pigmented prey (e.g. phytoplankton) that increases copepod susceptibility to visual predation (Juhl *et al.*, 1996; Tsuda *et al.*, 1998), instead of an heterotrophic, not pigmented protist. Still, Abrahamsen *et al.* (2010) reported that *M. norvegica* might rely more on hydromechanical signals than vision to detect active prey like copepods. Actually, *M. norvegica* conduct diel vertical migrations (Onsrud and Kaartvedt, 1998; Onsrud *et al.*, 2004) and most encounters between *M. norvegica* and copepods might take place at night when visual predation is limited. Particularly in the Gullmar Fjord, populations of *M. norvegica* remain deeper during the daytime and ascend to layers above 30 m only at night (Spicer and Strömberg, 2002). Thus, copepods may find more advantageous to modify their diel antipredator feeding behaviour upon the presence of other visual predators like fish that occur in upper, more illuminated layers during daylight hours (Øresland and André, 2008).

We found that seasonal photoperiod and prey availability had a significant influence on the diel feeding behaviour of copepods. The feeding rhythms were less pronounced in summer (16 h of light) than in autumn (10 h light). The diel rhythms of marine copepods are usually flexible over seasons (Båmstedt, 1984; Williams and Conway, 1984; Frost, 1988; Durbin *et al.*, 1995). Frost (1988) suggested that such variations between seasons are independent of prey availability, metabolic balance or thermal stratification, but driven by predation risk. In this regard, the seasonal photoperiod defines the relative time that copepods are exposed to a higher visual predation risk. As periods of higher predation vulnerability become relatively longer, copepods might diminish their antipredator behaviour to optimize the trade-off between eating and not being eaten (Lima and Bednekoff, 1999). Clearly, copepods must lower the intensity of their feeding rhythms when safe periods (i.e. dark periods) are too short for feeding to meet metabolic demands.

Regarding prey availability, the hunger/satiation hypothesis affirms that higher food availability normally results in larger amplitudes of vertical migration (Huntley and Brooks, 1982; Verheye and Field, 1992), which would enhance copepod feeding rhythms if food conditions in upper layers are better (Simard *et al.*, 1985; Peterson *et al.*, 1990; Besiktepe *et al.*, 2005). We found that the diel rhythms of *C. typicus* in the laboratory were more intense at low food concentrations. Low-food conditions decrease encounter rates with prey and copepods must swim for longer times and cover larger distances to feed (Saiz *et al.*, 1992), thus increasing their risk of being detected by predators (Uttieri *et al.*, 2013; Kiørboe *et al.*, 2014). Under these conditions, copepods would instead enhance their nocturnal feeding activity because foraging during the daylight hours would be too risky for them. We cannot strictly test this hypothesis since we only conducted one low-food experiment, but our findings are in agreement with those of Hassett and Blades-Eckelbarger (1995), who found that the diel feeding cycles of *Acartia tonsa* were more pronounced in the low-food treatment. In contrast, Calbet *et al.* (1999) reported that at lower food concentrations the diel feeding rhythms of *C. typicus* remained invariable and those of *A. grani* even vanished. Hence, the effect of food availability on copepod nocturnal feeding remains unclear and might be species-specific and/or depend on environmentally determined previous life history.

## CONCLUSIONS

Our study is one of the few works that addressed direct effects of predators on copepod feeding rhythms using freely swimming predators instead of predator exudates. Still, we did not detect any large effect of the predator *Meganyctiphanes norvegica* on the diel feeding behaviour of the marine calanoid *Centropages typicus*, whereas effects of other factors such as seasonal photoperiod and prey availability emerged. Therefore, the non-lethal direct effects of predators on the feeding activity of marine copepods might not be as relevant as in freshwater zooplankton, and it will require further effort to assess their role in plankton trophic interactions in marine systems.

## DATA ARCHIVING

Our data will be archived in a data repository after publication.

## References

[ref1] Abrahamsen M. B., Browman H. I., Fields D. M. and Skiftesvik A. B. (2010) The three-dimensional prey field of the northern krill, *Meganyctiphanes norvegica*, and the escape responses of their copepod prey. Mar. Biol., 157, 1251–1258. 24391246 10.1007/s00227-010-1405-9 PMC3873013

[ref2] Agersted M. D. and Nielsen T. G. (2016) Functional biology of sympatric krill species. J. Plankton Res., 38, 575–588.

[ref3] Agrawal A. A. (2001) Phenotypic plasticity in the interactions and evolution of species. Science, 294, 321–326. 11598291 10.1126/science.1060701

[ref4] Alcaraz M., Saiz E. and Calbet A. (2007) *Centropages* behaviour: swimming and vertical migration. Prog. Oceanogr., 72, 121–136.

[ref5] Atkinson A., Ward P., Williams R. and Poulet S. A. (1992) Feeding rates and diel vertical migration of copepods near South Georgia: comparison of shelf and oceanic sites. Mar. Biol., 114, 49–56.

[ref6] Atkinson A., Shreeve R. S., Pakhomov E. A., Priddle J., Blight S. P. and Ward P. (1996) Zooplankton response to a phytoplankton bloom near South Georgia, Antarctica. Mar. Ecol. Prog. Ser., 144, 195–210.

[ref7] Baars M. A. and Oosterhuis S. S. (1984) Diurnal feeding rhythms in North Sea copepods measured by gut fluorescence, digestive enzyme activity and grazing on labelled food. Neth. J. Sea Res., 18, 97–119.

[ref8] Båmstedt U. (1984) Diel variations in the nutritional physiology of *Calanus glacialis* from Lat. 78 ^o^N in the summer. Mar. Biol., 79, 257–267.

[ref9] Båmstedt U. and Karlson K. (1998) Euphausiid predation on copepods in coastal waters of the Northeast Atlantic. Mar. Ecol. Prog. Ser., 172, 149–168.

[ref10] Besiktepe S., Svetlichny L., Yuneva T., Romanova Z. and Shulman G. (2005) Diurnal gut pigment rhythm and metabolic rate of *Calanus euxinus* in the Black Sea. Mar. Biol., 146, 1189–1198.

[ref11] Besiktepe S. and Dam H. G. (2002) Coupling of ingestion and defecation as a function of diet in the calanoid copepod *Acartia tonsa*. Mar. Ecol. Prog. Ser., 229, 151–164.

[ref12] Beyer F. (1992) *Meganyctiphanes norvegica* (M. sars) (Euphausiacea) a voracious predator on *Calanus*, other copepods, and ctenophores, in Oslofjorden, southern Norway. Sarsia, 77, 189–206.

[ref13] Bochdansky A. B., Bollens S. M., Rollwagen-Bollens G. C. and Gibson A. H. (2010) Effect of the heterotrophic dinoflagellate *Oxyrrhis marina* and the copepod *Acartia tonsa* on vertical carbon flux in and around thin layers of the phytoflagellate *Isochrysis galbana*. Mar. Ecol. Prog. Ser., 402, 179–196.

[ref14] Bollens S. M., Rollwagen-Bollens G., Quenette J. A. and Bochdansky A. B. (2011) Cascading migrations and implications for vertical fluxes in pelagic ecosystems. J. Plankton Res., 33, 349–355.

[ref15] Bollens S. M. and Frost B. W. (1991) Diel vertical migration in zooplankton: rapid individual response to predators. J. Plankton Res., 13, 1359–1365.

[ref16] Bollens S. M. and Frost B. W. (1989) Predator-induced diel vertical migration in a planktonic copepod. J. Plankton Res., 11, 1047–1065.

[ref17] Bollens S. M. and Stearns D. E. (1992) Predator-induced changes in the diel feeding cycle of a planktonic copepod. J. Exp. Mar. Biol. Ecol., 156, 179–186.

[ref18] Bundy M. H., Gross T. F., Coughlin D. J. and Strickler J. R. (1993) Quantifying copepod searching efficiency using swimming pattern and perceptive ability. Bull. Mar. Sci., 53, 15–28.

[ref19] Buskey E. J., Lenz P. H. and Hartline D. K. (2011) Sensory perception, neurobiology, and behavioral adaptations for predator avoidance in planktonic copepods. Adapt. Behav., 20, 57–66.

[ref20] Calbet A., Saiz E., Irigoien X., Alcaraz M. and Trepat I. (1999) Food availability and diel feeding rhythms in the marine copepods *Acartia grani* and *Centropages typicus*. J. Plankton Res., 21, 1009–1015.

[ref21] Cieri M. D. and Stearns D. E. (1999) Reduction of grazing activity of two estuarine copepods in response to the exudate of a visual predator. Mar. Ecol. Prog. Ser., 177, 157–163.

[ref22] Dagg M. J., Frost B. W. and Newton J. (1998) Diel vertical migration and feeding in adult female *Calanus pacificus*, *Metridia lucens* and *Pseudocalanus newmani* during a spring bloom in Dabob Bay, a fjord in Washington USA. J. Mar. Syst., 15, 503–509.

[ref23] Durbin A. G., Durbin E. G. and Wlodarczyk E. (1990) Diel feeding behavior in the marine copepod *Acartia tonsa* in relation to food availability. Mar. Ecol. Prog. Ser., 68, 23–45.

[ref24] Durbin E. G., Campbell R. G., Gilman S. L. and Durbin A. G. (1995) Diel feeding behavior and ingestion rate in the copepod *Calanus finmarchicus* in the southern Gulf of Maine during late spring. Cont. Shelf Res., 15, 539–570.

[ref25] Duren L. A. van and Videler J. J. (1996) The trade-off between feeding, mate seeking and predator avoidance in copepods: behavioural responses to chemical cues. J. Plankton Res., 18, 805–818.

[ref26] Frost B. W. (1972) Effects of size and concentration of food particles on the feeding behavior of the marine planktonic copepod *Calanus pacificus*. Limnol. Oceanogr., 17, 805–815.

[ref27] Frost B. W. (1988) Variability and possible adaptive significance of diel vertical migration in *Calanus pacificus*, a planktonic marine copepod. Bull. Mar. Sci., 43, 675–694.

[ref28] Gonçalves R. J. and Kiørboe T. (2015) Perceiving the algae: how feeding-current feeding copepods detect their nonmotile prey. Limnol. Oceanogr., 60, 1286–1297.

[ref29] Guillard R. R. L. (1983) Culture of phytoplankton for feeding marine invertebrates In Berg C. J. Jr. (ed.), Culture of marine invertebrate animals: selected readings, Hutchinson Ross Publishing, Stroudsburg, PA, pp. 108–132 pp. 29–60.

[ref30] Hansen P. J. (1989) The red tide dinoflagellate *Alexandrium tamarense*: effects on behaviour and growth of a tintinnid ciliate. Mar. Ecol. Prog. Ser., 53, 105–116.

[ref31] Hassett R. P. and Blades-Eckelbarger P. (1995) Diel changes in gut-cell morphology and digestive activity of the marine copepod *Acartia tonsa*. Mar. Biol., 124, 59–69.

[ref32] Hayward T. L. (1980) Spatial and temporal feeding patterns of copepods from the North Pacific central gyre. Mar. Biol., 58, 295–309.

[ref33] Head E. J. H., Harris L. R. and Abou Debs C. (1985) Effect of daylength and food concentration on *in situ* diurnal feeding rhythms in Arctic copepods. Mar. Ecol. Prog. Ser., 24, 281–288.

[ref34] Heuschele J., Ceballos S., Andersen Borg C. M., Bjærke O., Isari S., Lasley-Rasher R., Lindehoff E., Souissi A. et al. (2014) Non-consumptive effects of predator presence on copepod reproduction: insights from a mesocosm experiment. Mar. Biol., 161, 1653–1666.

[ref35] Huntley M. and Brooks E. R. (1982) Effects of age and food availability on diel vertical migration of *Calanus pacificus*. Mar. Biol., 71, 23–31.

[ref36] Hwang J. S. and Strickler R. (2001) Can copepods differentiate prey from predator hydromechanically? Zool. Stud., 40, 1–6.

[ref37] Hylander S. and Hansson L.-A. (2013) Vertical distribution and pigmentation of Antarctic zooplankton determined by a blend of UV radiation, predation and food availability. Aquat. Ecol., 47, 467–480.

[ref38] Irigoien X., Head R., Klenke U., Meyer-Harms B., Harbour D., Niehoff B., Hirche H. J. and Harris R. (1998) A high frequency time series at weathership M, Norwegian Sea, during the 1997 spring bloom: feeding of adult female *Calanus finmarchicus*. Mar. Ecol. Prog. Ser., 172, 127–137.

[ref39] Irigoien X. (1998) Gut clearance rate constant, temperature and initial gut contents: a review. J. Plankton Res., 20, 997–1003.

[ref40] Juhl A. R., Ohman M. D. and Goericke R. (1996) Astaxanthin in *Calanus pacificus*: assessment of pigment-based measures of omnivory. Limnol. Oceanogr., 41, 1198–1207.

[ref41] Kaartvedt S., Røstad A., Fiksen Ø., Melle W., Torgersen T., Breien M. T. and Klevjer T. A. (2005) Piscivorous fish patrol krill swarms. Mar. Ecol. Prog. Ser., 299, 1–5.

[ref42] Kiørboe T., Saiz E., Tiselius P. and Andersen K. H. (2018) Adaptive feeding behavior and functional responses in zooplankton. Limnol. Oceanogr., 63, 308–321.

[ref43] Kiørboe T., Jiang H., Goncalves R. J., Nielsen L. T. and Wadhwa N. (2014) Flow disturbances generated by feeding and swimming zooplankton. Proc. Natl. Acad. Sci. U. S. A., 111, 11738–11743. 25071196 10.1073/pnas.1405260111 PMC4136613

[ref44] Kiørboe T., Saiz E. and Visser A. (1999) Hydrodynamic signal perception in the copepod *Acartia tonsa*. Mar. Ecol. Prog. Ser., 179, 97–111.

[ref45] Lasley-Rasher R. S. and Yen J. (2012) Predation risk suppresses mating success and offspring production in the coastal marine copepod *Eurytemora herdmani*. Limnol. Oceanogr., 57, 433–440.

[ref46] Lass S., Tarling G. A., Virtue P., Matthews J. B. L., Mayzaud P. and Buchholz F. (2001) On the food of northern krill *Meganyctiphanes norvegica* in relation to its vertical distribution. Mar. Ecol. Prog. Ser., 214, 177–200.

[ref47] Lima S. L. (1998) Nonlethal effects in the ecology of predator-prey interactions. Bioscience, 48, 25–34.

[ref48] Lima S. L. and Bednekoff P. A. (1999) Temporal variation in danger drives antipredator behavior: the predation risk allocation hypothesis. Am. Nat., 153, 649–659. 29585647 10.1086/303202

[ref49] Lima S. L. and Dill L. M. (1990) Behavioral decisions made under the risk of predation: a review and prospectus. Can. J. Zool., 68, 619–640.

[ref50] Longhurst A. R. (1985) The structure and evolution of plankton communities. Prog. Oceanogr., 15, 1–35.

[ref51] McClatchie S. (1985) Feeding behaviour in *Meganyctiphanes norvegica* (M. Sars) (Crustacea: Euphausiacea). J. Exp. Mar. Biol. Ecol., 86, 271–284.

[ref52] Nejstgaard J. C., Naustvoll L. J. and Sazhin A. (2001) Correcting for underestimation of microzooplankton grazing in bottle incubation experiments with mesozooplankton. Mar. Ecol. Prog. Ser., 221, 59–75.

[ref53] Nicol S. (1986) Shape, size and density of daytime surface swarms of the euphausiid *Meganyctiphanes norvegica* in the Bay of Fundy. J. Plankton Res., 8, 29–39.

[ref54] Ohman M. D. (1988) Behavioral responses of zooplankton to predation. Bull. Mar. Sci., 43, 530–550.

[ref55] Ohman M. D. (1990) The demographic benefits of diel vertical migration by zooplankton. Ecol. Monogr., 60, 257–281.

[ref56] Olivares M., Calbet A. and Saiz E. (2020) Effects of multigenerational rearing, ontogeny and predation threat on copepod feeding rhythms. Aquat. Ecol., 54, 697–709.

[ref57] Onsrud M. S. R., Kaartvedt S., Røstad A. and Klevjer T. A. (2004) Vertical distribution and feeding patterns in fish foraging on the krill *Meganyctiphanes norvegica*. ICES J. Mar. Sci., 61, 1278–1290.

[ref58] Onsrud M. S. R. and Kaartvedt S. (1998) Diel vertical migration of the krill *Meganyctiphanes norvegica* in relation to physical environment, food and predators. Mar. Ecol. Prog. Ser., 171, 209–219.

[ref59] Øresland V. and André C. (2008) Larval group differentiation in Atlantic cod (*Gadus morhua*) inside and outside the Gullmar Fjord. Fish. Res., 90, 9–16.

[ref60] Peterson W. T., Painting S. J. and Hutchings L. (1990) Diel variations in gut pigment content, diel vertical migration and estimates of grazing impact for copepods in the southern Benguela upwelling region in October 1987. J. Plankton Res., 12, 259–281.

[ref61] Preisser E. L., Bolnick D. I. and Benard M. F. (2005) Scared to death? The effects of intimidation and consumption in predator–prey interactions. Ecology, 86, 501–509.

[ref62] Runge J. A. (1988) Should we expect a relationship between primary production and fisheries? The role of copepod dynamics as a filter of trophic variability. Hydrobiologia, 167/168, 61–71.

[ref63] Saiz E., Alcaraz M. and Paffenhöfer G. A. (1992) Effects of small-scale turbulence on feeding rate and gross-growth efficiency of three *Acartia* species (Copepoda: Calanoida). J. Plankton Res., 14, 1085–1097.

[ref64] Saiz E., Tiselius P., Jonsson P. R., Verity P. and Paffenhöfer G. A. (1993) Experimental records of the effects of food patchiness and predation on egg production of *Acartia tonsa*. Limnol. Oceanogr., 38, 280–289.

[ref65] Schmidt K. (2010) Food and feeding in Northern krill (*Meganyctiphanes norvegica* Sars). Adv. Mar. Biol., 57, 127–171. 20955891 10.1016/B978-0-12-381308-4.00005-4

[ref66] Schmitz O. J., Krivan V. and Ovadia O. (2004) Trophic cascades: the primacy of trait-mediated indirect interactions. Ecol. Lett., 7, 153–163.

[ref67] Schneider C. A., Rasband W. S. and Eliceiri K. W. (2012) NIH image to ImageJ: 25 years of image analysis. Nat. Methods, 9, 671–675. 22930834 10.1038/nmeth.2089 PMC5554542

[ref68] Sih A. (1980) Optimal behavior: can foragers balance two conflicting demands? Science, 210, 1041–1043. 17797495 10.1126/science.210.4473.1041

[ref69] Simard Y., Lacroix G. and Legendre L. (1985) In situ twilight grazing rhythm during diel vertical migrations of a scattering layer of *Calanus finmarchicus*. Limnol. Oceanogr., 30, 598–606.

[ref70] Simard Y. and Harvey M. (2010) Predation on Northern krill (*Meganyctiphanes norvegica* Sars). Adv. Mar. Biol., 57, 277–306. 20955896 10.1016/B978-0-12-381308-4.00010-8

[ref71] Someren Gréve H. van, Kiørboe T. and Almeda R. (2019) Bottom-up behaviourally mediated trophic cascades in plankton food webs. Proc. R. Soc. B, 286, 20181664. 10.1098/rspb.2018.1664 PMC6408594 30963919

[ref72] Spicer J. I. and Strömberg J. O. (2002) Diel vertical migration and the haemocyanin of krill *Meganyctiphanes norvegica*. Mar. Ecol. Prog. Ser., 238, 153–162.

[ref73] Suca J. J., Pringle J. W., Knorek Z. R., Hamilton S. L., Richardson D. E. and Llopiz J. K. (2018) Feeding dynamics of Northwest Atlantic small pelagic fishes. Prog. Oceanogr., 165, 52–62.

[ref74] Tarling G. A., Jarvis T., Emsley S. M. and Matthews J. B. L. (2002) Midnight sinking behaviour in *Calanus finmarchicus*: a response to satiation or krill predation? Mar. Ecol. Prog. Ser., 240, 183–194.

[ref75] Tarling G. A., Matthews J. B. L., Saborowski R. and Buchholz F. (1998) Vertical migratory behaviour of the euphausiid, *Meganyctiphanes norvegica*, and its dispersion in the Kattegat Channel. Hydrobiologia, 375/376, 331–341.

[ref76] Tiselius P., Jonsson P. R., Kuurtvedt S., Olsen E. M. and Jørstud T. (1997) Effects of copepod foraging behavior on predation risk: an experimental study of the predatory copepod *Pareuchaeta norvegica* feeding on *Acartia clausi* and *A. tonsa* (Copepoda). Limnol. Oceanogr., 42, 164–170.

[ref77] Tiselius P. and Jonsson P. (1990) Foraging behaviour of six calanoid copepods: observations and hydrodynamic analysis. Mar. Ecol. Prog. Ser., 66, 23–33.

[ref78] Tönnesson K. and Tiselius P. (2005) Diet of the chaetognaths *Sagitta setosa* and *S. elegans* in relation to prey abundance and vertical distribution. Mar. Ecol. Prog. Ser., 289, 177–190.

[ref79] Torgersen T. (2001) Visual predation by the euphausiid *Meganyctiphanes norvegica*. Mar. Ecol. Prog. Ser., 209, 295–299.

[ref80] Tsuda A., Saito H. and Hirose T. (1998) Effect of gut content on the vulnerability of copepods to visual predation. Limnol. Oceanogr., 43, 1944–1947.

[ref81] Uttieri M., Cianelli D. and Zambianchi E. (2013) Behaviour-dependent predation risk in swimming zooplankters. Zool. Stud., 52, 32.

[ref82] Vargas C. A., Tönnesson K., Sell A., Maar M., Møller E. F., Zervoudaki T., Giannakourou A., Christou E. et al. (2002) Importance of copepods versus appendicularians in vertical carbon fluxes in a Swedish fjord. Mar. Ecol. Prog. Ser., 241, 125–138.

[ref83] Verheye H. M. and Field J. G. (1992) Vertical distribution and diel vertical migration of *Calanoides carinatus* (Krøyer, 1849) developmental stages in the southern Benguela upwelling region. J. Exp. Mar. Biol. Ecol., 158, 123–140.

[ref84] Visser A. W. (2007) Motility of zooplankton: fitness, foraging and predation. J. Plankton Res., 29, 447–461.

[ref85] Werner E. E. and Peacor S. D. (2003) A review of trait-mediated indirect interactions in ecological communities. Ecology, 84, 1083–1100.

[ref86] Williams R. and Conway D. V. P. (1984) Vertical distribution, and seasonal and diurnal migration of *Calanus helgolandicus* in the Celtic Sea. Mar. Biol., 79, 63–73.

[ref87] Youngbluth M. J., Bailey T. G., Davoll P. J., Jacoby C. A., Blades-Eckelbarger P. I. and Griswold C. A. (1989) Fecal pellet production and diel migratory behavior by the euphausiid *Meganyctiphanes norvegica* effect benthic-pelagic coupling. Deep-Sea Res., 36, 1491–1501.

